# PolySac3DB: an annotated data base of 3 dimensional structures of polysaccharides

**DOI:** 10.1186/1471-2105-13-302

**Published:** 2012-11-14

**Authors:** Anita Sarkar, Serge Pérez

**Affiliations:** 1 Centre de Recherches sur les Macromolécules Végétales (CERMAV*) Centre National de la Recherche Scientifique, Grenoble Cedex 9, BP 53X, F-38041, France; 2 Université Joseph Fourier, Grenoble, France; 3 European Synchrotron Radiation Facility, (ESRF), Grenoble, France

**Keywords:** Polysaccharides, Carbohydrates, Three-dimensional (3D) database, Graphical user interface (GUI), Atomic coordinates, Information portal

## Abstract

**Background:**

Polysaccharides are ubiquitously present in the living world. Their structural versatility makes them important and interesting components in numerous biological and technological processes ranging from structural stabilization to a variety of immunologically important molecular recognition events. The knowledge of polysaccharide three-dimensional (3D) structure is important in studying carbohydrate-mediated host-pathogen interactions, interactions with other bio-macromolecules, drug design and vaccine development as well as material science applications or production of bio-ethanol.

**Description:**

PolySac3DB is an annotated database that contains the 3D structural information of 157 polysaccharide entries that have been collected from an extensive screening of scientific literature. They have been systematically organized using standard names in the field of carbohydrate research into 18 categories representing polysaccharide families. Structure-related information includes the saccharides making up the repeat unit(s) and their glycosidic linkages, the expanded 3D representation of the repeat unit, unit cell dimensions and space group, helix type, diffraction diagram(s) (when applicable), experimental and/or simulation methods used for structure description, link to the abstract of the publication, reference and the atomic coordinate files for visualization and download. The database is accompanied by a user-friendly graphical user interface (GUI). It features interactive displays of polysaccharide structures and customized search options for beginners and experts, respectively. The site also serves as an information portal for polysaccharide structure determination techniques. The web-interface also references external links where other carbohydrate-related resources are available.

**Conclusion:**

PolySac3DB is established to maintain information on the detailed 3D structures of polysaccharides. All the data and features are available via the web-interface utilizing the search engine and can be accessed at http://polysac3db.cermav.cnrs.fr.

## Background

Carbohydrates are an essential class of biological molecules. They are ubiquitous in the living world, occurring mostly as polysaccharides and oligosaccharides, frequently in the form of conjugates with other bio-molecules like proteins (glycoproteins) or lipids (glycolipids). In comparison to the more vastly studied nucleic acids and proteins, carbohydrates have an information carrying capacity of a much higher degree by virtue of the presence of a multiplicity of chiral centers, combinations of various glycosidic linkages and a large number of functional group modifications that might include acetylation, methylation, oxidation and sulfation, creating an even greater diversity out of the already numerous possible building blocks (monosaccharides) [1]. Polysaccharides (or carbohydrate polymers) are macromolecules made up of repeating monosaccharide units linked by glycosidic bonds. They are essential cellular constituents and their roles extend far beyond being mere energy stores (e.g. starch and glycogen) and structural support agents (e.g. cellulose and chitin). They partake in regulating cell wall plasticity (e.g. pectins, alginates and carrageenans), cell signaling, governing solution properties of some physiological fluids and participating in the structural build-up of the inter-cellular matrix (e.g. glycosaminoglycans), eliciting immune responses, cancer progression and as an anti-coagulating agent for the prevention of blood clots (e.g. heparin). Polysaccharides are useful in tissue engineering and repair, wound healing and drug delivery systems, biofuels, biodegradable fibers and bio-composites, due to their generally non-toxic and biodegradable properties and being a renewable resource. Polysaccharides are frequently found on the cell surface of single-celled or multicellular organisms [2] and in the extra-cellular matrix of eukaryotes [3] and are involved in host-pathogen recognition events.

Polysaccharides range in structure from linear to highly-branched. They are often quite heterogeneous, containing slight modifications of their repeating units. This high degree of complexity and inherent micro-heterogeneity of polysaccharide structures make them very difficult macromolecules to handle and explore experimentally. Their structure determines their properties and consequently their function. To understand the molecular basis of the native arrangements of polysaccharides and relating their properties and functions to their structures, the different levels of their structural organization must be determined. As with other macromolecules, the elucidation of the primary structure (implying the sequence of monomeric units with the respective glycosidic linkages) is a pre-requisite. Depending on their primary structures and biosynthesis, polysaccharides may have single or multiple chains in characteristic helical forms that define their secondary structure. Energetically favored interactions between chains of well-defined secondary structures may result in ordered organizations, referred to as tertiary structures. A higher level of organization involving further associations between these well-structured entities results in quaternary structures.

The most important method for the 3D structural characterization of crystalline (or semi-crystalline) polysaccharides is X-ray fiber diffraction. In contrast to other bio-macromolecules, the diffraction data that can be obtained from fibrillar samples of polysaccharides are not of a sufficient quantity and quality to provide enough experimental information to resolve the crystallographic structure unambiguously. A modeling technique must be used which allows the calculation of diffraction intensities from various models for comparison with the observed intensities. In general, the proposed models provide an accuracy of the final atomic coordinates within a few tenths of an Angstrom; typically the R values are around 0.20 in a majority of reported polysaccharide structures. Nuclear magnetic resonance (NMR) is used in assessing 3D features of polysaccharides in solution which adopt a more or less coiled structure, that fluctuate between local and overall conformations. These features can be translated into 3D structures with the use of molecular modeling. Indeed, molecular modeling has also become an essential component, not only as a complementary technique to be used in the elucidation of 3D crystalline structures, but also as a powerful tool in studying packing of polysaccharides that enables building of models, studying chain-chain interactions [4] and calculating energies. These molecular modeling techniques can be used to construct structures starting from the content of the crystallographic unit cell to much larger macromolecular assemblies offering a unique possibility to visualize morphological features which are in many cases, the relevant level of structural organization with respect to functions or properties of carbohydrate polymers.

3D structures provide information that is indispensable in many aspects of molecular interaction studies. The unification of the resources on carbohydrate polymers, combination of the various tools developed in this field and the free and simple availability of the results that have been generated are necessary for the advancement of polysaccharide science. Bioinformatics has played a role in unifying the resources and information available in genetics and proteomics. Similarly, glycoinformatics has a crucial role to play in the field of carbohydrates. Although a large amount of 3D information regarding the structure of polysaccharides has accumulated over time, the effort to collect, curate and disseminate this data electronically and freely to the scientific community has been feeble. PDB [5] contains very low number of polysaccharide entries, though some coordinates are available, and the Cambridge Structural Database [6] is not a free resource. The only similar contribution, with respect to polysaccharides, has been in the form of a book chapter published in 1997, wherein all the atomic coordinates of polysaccharide structures established by X-ray fiber diffraction have been reported and categorized [7]. A similar effort has been made for celluloses and cellulose derivatives in a book devoted to the structures of this important polysaccharide [8].

Here we report the construction of an annotated polysaccharide 3D structural database called PolySac3DB which provides details of experimental and modeled structures of polysaccharides.

## Construction and content

### Construction

PolySac3DB is a web-based, platform-independent, manually curated database of polysaccharide 3D structures. It currently runs on an Apache web server [9] hosted at Centre de Recherches sur les Macromolécules Végétales (CERMAV) with the application program Hypertext Preprocessor (PHP) [10]. It has been developed based on a combination of three layers. The underlying layer is the MySQL database system [11], a relational database management system [MySQL 5.1.41 (Community Server) with PBXT engine 1.0.09-rc] that stores all the structural information along with the respective publications in the back-end and provides the facility to link two or more tables in the database. The intermediate layer is an Apache-PHP application [Apache 2.x; PHP 5.3.1] that receives the query from the user and connects to the database to fetch data from the upper layer, which comprises populated HTML and PHP pages, to the web browser client. The PHP and Java scripts are embedded in the HTML web pages for this effect and are used as application programs for integrating the back-end (MySQL database) to the web pages (HTML). Apache is used as the web server for building the interface between the web browser and the application programs. HTML and PHP have been used to build the web interface.

### Content

#### Data sources –screening, conversion and information extraction

**Figure 1:**
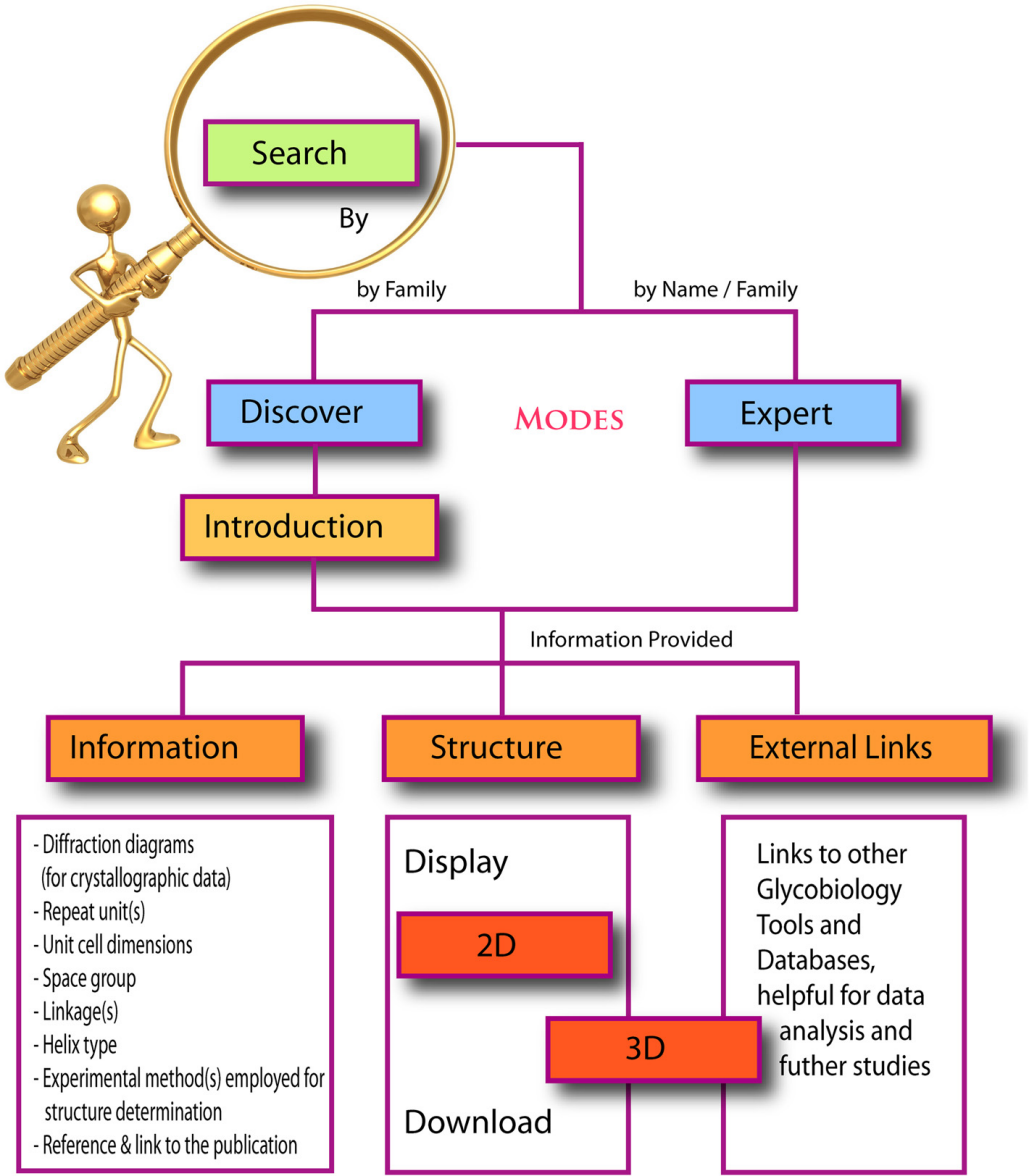
Schematic overview of the PolySac3DB organization and content.

In order to collect structural information about the constituent members of the various polysaccharide families, an extensive screening of literature was performed. This yielded 84 publications that supplied records of the atomic coordinates of polysaccharide (unit) structures established using various structure determination techniques as well as molecular modeling, predominantly containing diffraction data. Enough information could be extracted from these publications to fit the minimum information criteria set for this database and thereafter a total of 157 polysaccharide structures were incorporated into PolySac3DB. The classification of the polysaccharide structures into families is presented in Table 1. The information was manually extracted and curated before incorporation into the repository. The publications provided atomic coordinates within the asymmetric unit of the cell content available as fractional, Cartesian or cylindrical polar coordinates. The available data was converted to either fractional or Cartesian coordinates to generate the atomic coordinate files in standardized representations of PDB (Protein Data Bank) [5] or Mol2 (SYBYL) [12] formats. The files were generated using an in-house PHP script called PDBGenerator, developed for the construction of this database, which can convert fractional and cylindrical/polar coordinates to PDB format. Besides, SYBYL, PyMol, Mercury and Polys were also used to generate helical/expanded forms of the unit cell structures [12,14,15]. The afore-mentioned formats were chosen to provide a broad readability by various visualization programs as well as to expedite comparisons of glycan with nucleic acid and protein structure as well as computer simulation of their interactions. Application of the symmetry operators of the space groups was done to generate the atomic content of the unit cell and extend them to larger structures. Where symmetry operator information was unavailable, models were generated (wherever possible) to offer a representation of the expanded forms assumed by the polysaccharides. The 3D structures of the repeat units and the packing structures were split into two separate tables on the relational database, respectively. The work flow is described in Figure 1.

**Table 1:** Polysaccharide Families and their constituent members present in PolySac3DB

**No.**	**Family Name**	**Polysaccharide Member**	**Reference**
1	Agaroses	Agarose (single)	[16]
		Agarose (double)	[17]
		Agarose Molecular Models	[18]
2	Alginates	Poly-α-L-Guluronic Acid	[19]
		Poly-β-D-Mannuronic Acid	[20]
		Alginate Molecular Models	[21,22]
3	Amyloses & Starch	A Starch	[23,24]
		Starch Nanocrystals	[25]
		Amylopectins	[26]
		B Starch	[27]
		Amylose DMSO	[28]
		Amylose KOH	[29]
		Amylose Triacetate	[30]
		Amylose tri-O-ethyl (TEA3)	[31]
		Amylose V	[32]
		Amylose V propanol complex	[33]
4	Bacterial Polysaccharides	Dextran (high T polymorph)	[34]
		Dextran (low T polymorph)	[35]
		Exo-polysaccharide (*Burkholderia cepacia*)	[36]
		α (2-8)-linked Sialic Acid Polysaccharide	[37]
		M41 Capsular Polysaccharide (*E. coli*)	[38]
		O-antigenic polysaccharide (*E. coli* 1303)	Manuscript in preparation
		O-antigenic polysaccharide (*E. coli* O5ab)	Manuscript in preparation
		O-antigenic polysaccharide (*E. coli* O5ac)	Manuscript in preparation
		O-antigenic polysaccharide (*E. coli* O65)	Manuscript in preparation
		Capsular Polysaccharide (*Rhizobium trifolii*)	[39]
		Gellan Native K	[40]
		Gellan K	[41]
		Gellan Li	[42]
		RMDP17	[43]
		Welan (Ca)	[44]
		Xanthan	[45]
5	Carrageenans	Iota Carrageenan	[46]
		Iota Carrageenan (Na salt)	[47]
		Kappa Carrageenan	[48]
6	Celluloses	Cellulose I α	[49]
		Cellulose I β	[50]
		Cellulose I triacetate	[51]
		Cellulose II	[52]
		Cellulose II hydrate	[53]
		Cellulose II hydrazine	[54]
		Cellulose II triacetate	[55]
		Cellulose III_I_	[56]
		Cellulose IV_I_	[57]
		Cellulose microfibrils	[58]
7	Chitins & Chitosans	Chitin I (Chitin β)	[59]
		Chitin II (Chitin α)	[60]
		Chitosan (anhydrous)	[61]
		Chitosan (high T Polymorph)	[62]
8	Curdlans	Curdlan I (Native)	[63]
		Curdlan II	[64]
		Curdlan III	[65]
9	GAGs	Chondroitin (unsulphated)	[66]
		Chondroitin 4-sulphate Ca	[67]
		Chondroitin 4-sulphate K	[68]
		Chondroitin 4-sulphate Na	[69]
		Dermatan 4-sulphate Na (allomorphs I, II, III)	[70]
		Hyaluronate I & II Sodium	[71]
		Hyaluronate III Sodium	[72]
		Hyaluronate I Potassium	[73]
		Hyaluronate II Potassium	[74]
		Hyaluronate III Potassium	[75]
		Hyaluronate Calcium	[76]
		Hyaluronic acid	[77]
		Heparin (dp 12) Heparin (dp 18, 24, 30, 36)	[78,79]
		Keratan-6-sulphate	[80]
10	Galactoglucans	Galactoglucan	[81]
11	Galactomannans	Galactomannan	[82]
12	Glucomannans	Konjac glucomannan	[83]
13	Mannans	Mannan I	[84]
		Mannan II	[85]
		α-D-1,3-Mannan	[86]
		Mannan dihydrate	[87]
14	Pectins	Pectic Acid	[88]
		Calcium Pectate	[89]
		Sodium Pectate	[88]
		Polyuronides Molecular Models	[21,22]
		Arabinan	[90]
		Arabino-Galactan Type I	[90]
		Arabino-Galactan Type II	[90]
		RG-I	[90]
		RG-II	[91]
15	Scleroglucans	Scleroglucan	[92]
16	Xylans	Xylan (β-1,3)	[93]
		Xylan (β-1,4)	[94,95]
17	Nigeran	Nigeran	[96]
18	Others	Inulin hemihydrate	[97]
		Inulin monohydrate	[97]
		α-D-glucan	[98]
		α-1,3-glucan triacetate	[99]

The extracted data also included information about carbohydrate composition, glycosidic linkages as well as space group, unit cell dimensions (*a*, *b*, *c,* α, β and γ), the type of helix (that the polysaccharide chains form) which is made available via the ‘Expert Mode’. The experimental methods used in structure determination of the respective polysaccharide, the link to the abstract and the reference to the publication cited were also extracted. Particular attention was given to the recording of the available diffraction patterns, which are indeed the original experimental data from which the 3D structures were established. In the present version of PolySac3DB more than 120 diffractograms have been collected; they form a unique collection of information that have been generated over almost half a century of structural research in the area of carbohydrate polymers.

#### Data storage

Efficiency of data storage and management are the hallmarks of a fully-functional database. At present the database comprises four tables stored within the relational database working in the back-end of PolySac3DB developed using MySQL which provides the facility of linking/relating two or more tables in a database. The important tables within the database are ‘strucdata’, ‘images’, ‘polysac3dview’ and ‘polysac3d-dwnld’ that incorporate information regarding the experimental or modeled structures and other information extracted from the publications, the diffraction data and the figure legends and the atomic coordinates of the 3D structures for viewing and download, respectively. The tables are linked via a unique key to maintain non-redundancy in PolySac3DB. Subsequent tables can easily be added and logically connected to the existing relational database to accommodate more data about the polysaccharide structures that would be deemed relevant in the future.

## Utility and Discussion

### PolySac3DB search: Navigation and retrieval

The links to access various utilities and the search engine are provided on the left panel of the website via which the data content of the repository can be browsed and retrieved by the user. The ‘User Guide’ describes each search parameter and its output with detailed examples. A ‘Discover Mode’ is available that provides background information about the entry/family (mainly regarding occurrence, biosynthesis, property and function). The two-dimensional representations of the polysaccharide repeating unit have been constructed and made available through the ‘Discover Mode’ in PolySac3DB to aid users to find a familiar representation of the glycan. Information about the nature of the helical structure and all other information can be retrieved upon querying through the ‘Expert Mode’.

### Data access

**Figure 2:**
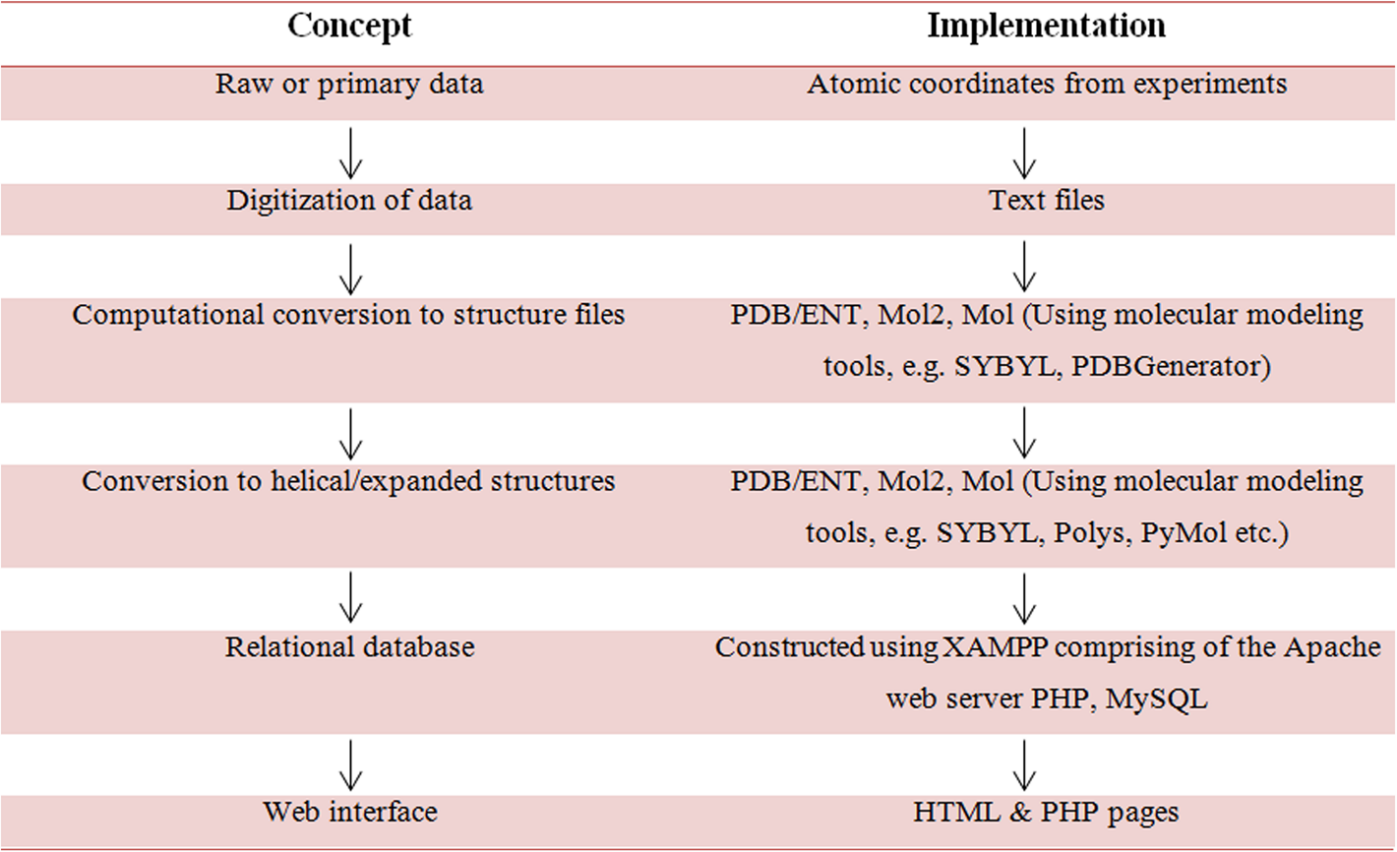
Workflow of the informatics tools used for PolySac3DB.

Data retrieval and usability are the primary goals set by the developers of an effective database. An interactive front-end was designed for PolySac3DB with HTML pages and server side scripts that extract data from the tables on the relational database for user-queries on ‘Search’ and display the retrieved information in a coherent manner. PolySac3DB is equipped with a user-friendly GUI for quick and easy access to the required data. The interface provides the user with options to search by ‘Name’ or ‘Family’ of the polysaccharide. This GUI was tested on different versions of four web browser clients (Google Chrome, Mozilla Firefox, Safari and Internet Explorer) with which it performed efficiently. The ‘User Guide’ gives a detailed description of the content and searchable options within the repository. A schematic over-view is provided in Figure 2. PolySac3DB also provides an overview of the polysaccharide structure determination methods, acting as an information portal on how X-ray, neutron and electron diffraction as well as molecular modeling are applied to polysaccharides. A list of references is provided on the site on a separate web-page incorporating all the publications from which the atomic coordinates of the structures in the database have been derived, besides proper referencing on the individual ‘Expert’ pages. In an effort to assimilate other relevant resources for sugars, ‘External Links’ are provided that empowers the user to explore more online glycoinformatics resources.

### PolySac3DB output

The bulk of the structure information for the polysaccharide entries is made available via the ‘Expert Mode’. 3D structures can be viewed over the website via the Jmol application [100]. Jmol is an interactive web browser applet that is an open-source, cross-platform 3D Java visualizing tool for visualizing chemical and molecular structures. It provides high-performance 3D rendering with standard available hardware. Downloading the atomic coordinates for further independent use is of course another option provided via the expert mode. The GUI has been designed to retrieve, interpret and display the related information about each entry stored in the back-end on the four tables of the relational database and display it interactively to the user.

Data arrangement followed data collection and fields were set up under which the data would be categorized in the database. Since the majority of experimental structures in our dataset contained entries from crystallography, the data fields were defined upon these guidelines.

### Beyond the unit cell contents

**Figure 3:**
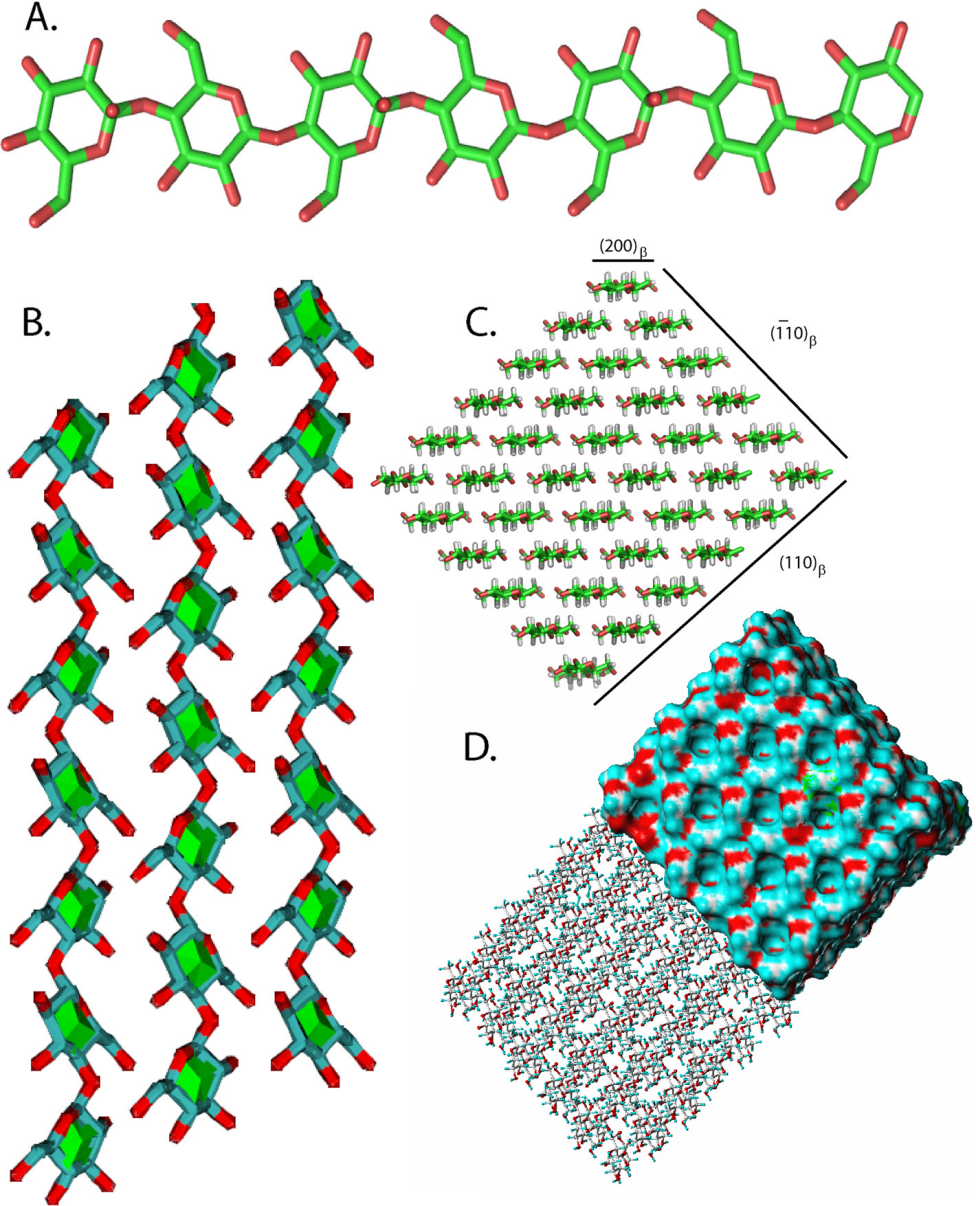
**Cellulose chain conformation and morphology.** (**A**) Crystalline conformations of the cellulose chain in the 1β allomorph showing the disordered orientation of hydroxylic hydrogen atoms. (**B**) Relative orientation of cellulose chains of native cellulose 1β. (**C**) Molecular model of the microfibril of cellulose projected along the fibril axis along with the indexing of the surfaces. (**D**) Computer representation of the crystalline morphology and surfaces of the microfibril of cellulose made up of 36 cellulose chains.

Besides providing essential structural information, the 3D crystallographic data on polysaccharides open the way to further insights into other strata of structural organization. The following describes some examples of such extensions. In the case of celluloses, the availability of an accurate description of the crystalline structures of the two allomorphs (cellulose Iα [49] and cellulose Iβ [50] has provided new insights into the crystalline morphology of the native celluloses. These models were used to generate different ordered atomic surfaces, and evaluate their occurrence along with their respective features. Full atomic models of the crystalline morphology and surfaces of a micro-fibril of cellulose made up of 36 cellulose chains could be conceptualized [58]. Such a model was built as a part of the present database as shown in Figure 3.

**Figure 4:**
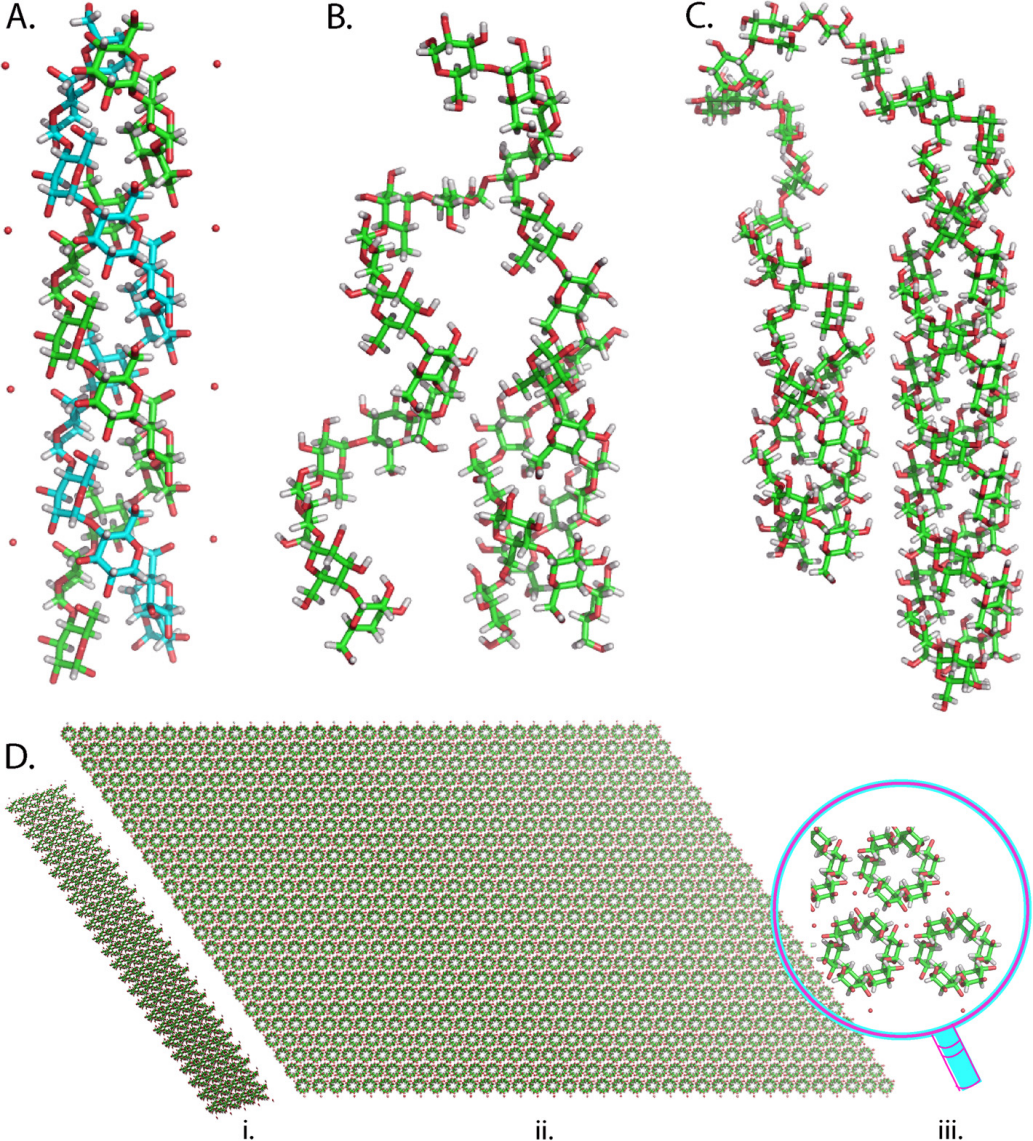
**Different levels of structural organization in starch.** (**A**) Representation of the left-handed single chains that are parallel stranded in A-starch double helix. (**B**) and (**C**) Representations of the double helix of crystalline starch after modeling the branching point between the strands. (**D**) Computer representation of an ideal platelet nanocrystal showing (i) width of the platelet with the tilt angle of the double helical component, (ii) composition of the platelet and (iii) the enlarged view of the constituent repeating unit.

In the case of starch, full atomic models of a nano-crystal containing 300 double helical segments in full crystallographic register have been constructed as a part of the work on PolySac3DB. They explain the morphology of these macromolecular assemblies as revealed by transmission electron microscopy [25]. Figure 4 displays the different levels of structural organizations of starch as represented in the various structures present in PolySac3DB.

Cases occur where the quality of the experimental data are far from being sufficient to establish a non-ambiguous model of the 3D arrangement. For example, extensive molecular modeling has provided insights about the way chain-pairing occurs, being mediated by Ca^2+^ interactions in alginates and pectins [22].

## Conclusion

The aim of the present work was to provide an organization of all polysaccharide atomic coordinates in one single database serving as a unifying repository and to categorize them in a logical fashion for the user to access the required data using pre-customized searching techniques. The search period covered by the present investigation is about 50 years, during which these structural models have been proposed in carbohydrate research. In view of the crucial role played by molecular modeling techniques, it was important to preserve, organize and distribute the macromolecular models developed. Their extensions to higher level of structures may expand our knowledge from the molecular to the microscopic level and help scrutinizing the several levels of structural organization of polysaccharides that underline their remarkable functions and properties. At a time when more and more carbohydrates and especially polysaccharides are being called to the fore for their increased use in a plethora of areas as diversified as tissue engineering and repair, wound healing, drug delivery systems, biofuels, bio-degradable fibers and bio-composites due to their generally non-toxic and biodegradable properties and being a renewable resource, PolySac3DB shall be an asset to the community for probing further into the behavior of this class of biological macromolecules.

## Availability and requirements

The database PolySac3DB is now available at http://polysac3db.cermav.cnrs.fr under the glycoscience portal : glyco3d.cermav.cnrs.fr

## Abbreviations

3D: Three-dimensional; GUI: Graphical user interface; NMR: Nuclear magnetic resonance; PDB: Protein data bank.

## Competing interests

The authors declare that they have no competing interests.

## Authors’ contributions

Corresponding author SP designed the framework for the project and wrote the detailed ‘Discover’ notes. AS designed the method, developed the MySQL database, web interface and related PHP scripts and wrote the manuscript. All authors have read and approved the final manuscript.
